# Global expansion of *Vibrio* spp. in hot water

**DOI:** 10.1111/1758-2229.13135

**Published:** 2022-12-15

**Authors:** Luigi Vezzulli

**Affiliations:** ^1^ Department of Earth, Environmental and Life Sciences (DISTAV) University of Genoa Genoa Italy

Climate change is a global phenomenon which is affecting marine and terrestrial environments worldwide. According to IPCC global warming defined as an increase in combined surface air and sea surface temperatures averaged over the globe and over a 30‐year period reached approximately 1°C (likely between 0.8 and 1.2°C) above pre‐industrial levels in 2017, increasing at 0.2°C (likely between 0.1 and 0.3°C) per decade (Allen et al., 2018). The surface of the ocean has warmed about one degree during the last 80 years and the rate of warming in the top 2000 m of the ocean over the past few decades was about 40 percent higher than previously estimated (US EPA, 2016). Recent projections suggest that ocean surface (0–2000 m) is likely to warm by 1546–2170 ZJ relative to 2005–2019, corresponding to 17–26 cm sea‐level rise from thermal expansion (Lyu et al., 2021).

Changing climatic conditions under such dramatic scenario are becoming increasingly suitable for the transmission of several infectious diseases caused by microbial pathogens, by directly affecting their biological features (e.g., growth, survival, and virulence), reservoirs and vectors, or by favouring their transmission through induced changes in ecosystems and human behaviour (Mora et al., 2022). In aquatic environments bacterial belonging to the *Vibrio* genus tend to be more common in warmer waters, especially above 17°C, and are particularly sensitive to changing environmental conditions (Vezzulli et al., 2016). This bacterial genus contains more than 100 confirmed species (http://www.bacterio.net/vibrio.html), 12 of which have been demonstrated to cause infections in humans. *Vibrio cholerae* the causative agent of cholera is by far the most relevant *Vibrio* species of public health concern accounting for about three million cases of human infections each year, with a case fatality rate of about 2.4% (Ali et al., 2015). However, only O1 and O139 serogroups are associated with epidemic cholera whilst *V. cholerae* serogroups other than O1 and O139 also designated non‐toxigenic *V. cholerae* (NTVC) are not associated with the cholera disease. Other non‐cholera *Vibrio* species of relevance for human health include *Vibrio parahaemolyticus*, *Vibrio vulnificus*, and *Vibrio alginolyticus*. These microorganisms can cause illness that may range in severity from mild (e.g., gastroenteritis, skin wound, etc.) to life‐threatening (e.g., necrotizing fasciitis) generally transmitted via ingestion of contaminated water or food or to exposure of skin wounds to aquatic environments and animals (Ceccarelli et al., 2019).

Non‐cholera vibrios and their associated infections have been reported to increase due to global warming (Baker‐Austin et al., 2018; Trinanes & Martinez‐Urtaza., 2021). In 1989, the Centres for Disease Control and Prevention (CDC) have established COVIS, the Cholera and Other *Vibrio* Illness Surveillance system, for reporting human infections with pathogenic species of the family *Vibrionaceae*, which cause vibriosis and cholera (https://www.cdc.gov/vibrio/surveillance.html). According to COVIS the number of *Vibrio* cases in recent years has been found to increase in parallel with the rise of SST (Newton et al., 2012). A very similar trend has been reported for Europe, especially in the Baltic Sea, in relation to unprecedented rate of warming of this area (Baker‐Austin et al., 2016). Overall, *Vibrio* species have undergone a global expansion over the past few decades reaching new areas of the world that were previously considered adverse for these organisms (Trinanes & Martinez‐Urtaza., 2021). Recent projections showed that coastal areas suitable for *Vibrio* could cover 38,000 km of new coastal areas by 2100 under the most unfavourable climate scenario with an expansion rate of season suitability in these regions of around 1 month every 30 years (Trinanes & Martinez‐Urtaza., 2021).

According to surveillance data NTVC strains prevailed, among other pathogenic *Vibrio* spp., with infections showing a steady increase over the last two decades in parallel to the intensity and extension of warming events in the United States and Europe (Vezzulli et al., 2020). As striking examples of such expansion during the 2014 heatwave NTVC infections were identified across coastal areas of Sweden and Finland, with cases reaching subarctic regions as far north as 65 N (160 km from the Arctic Circle) (Baker‐Austin et al., 2016). In March 2018, cases of NTVC infections were reported among local populations in Vancouver Island, British Columbia (Canada) the very first report of NTVC infections in this region (Swinkels & Waters, 2018). Adding to these reports, the study from Rehm and colleagues published in Environmental Microbiology Reports, provide first evidence on the presence of NTVC strains in Serbian lakes and ponds suggesting NTVC expansion may also occur in inland waters (Rehm et al., 2022). Notably NTVC strains were found in most of the water bodies investigated in their study, at concentrations ranging from 5.4 × 10^1^ to 1.86 × 10^4^ CFU 100 ml^−1^ which are consistent with those found during NTVC infections in brackish and marine waters (Vezzulli et al., 2020). These findings add to the accumulating evidence that although generally neglected, NTVC strains and related infections are on the rise representing one of the most striking examples of emerging human diseases linked to climate change. These strains are highly diverse genetically and there are more routes by which they cause human disease including consumption of oysters, clams, or exposure to seawater or freshwater in lakes and rivers (Aydanian et al., 2011). NTVC do not normally carry the cholera toxin (CT) but may produce several other toxins such as hemolysins, RTX toxins, cholix toxin and type III secretion system, that are generally associated with self‐limited gastroenteritis or mild to severe extra‐intestinal symptoms (Schwartz et al., 2019). NTVC strains may thus act as reservoirs of virulence genes for toxigenic strains in the aquatic environment and could contribute to the emergence of new pathogenic strains through horizontal gene transfer, including strains with epidemic potential (Faruque et al., 2004). Notably, fatal cases of necrotizing fasciitis (a life‐threatening infection of skin and fascia that generally shows rapid progression causing extensive necrosis) and septicemia associated with NTVC have increasingly been reported (Vezzulli et al., 2020).

Overall, despite NTVC (and more generally non‐cholera vibrio) infections are on the rise globally due to climate change such diseases are often not diagnosed or reported. An explanation for this lies in the fact that clinicians are generally inexperience in suspecting vibrios as possible cause of human infection, and to the fact that diagnostic laboratories may not be trained to apply appropriate enrichment and culture media to isolate these bacteria (Baker‐Austin et al., 2017). Raising awareness of *Vibrio* diseases among medical practitioners and implementation of surveillance systems (such as the COVIS system in the United States), in countries that are facing an increasing number of cases (e.g., Northern Europe), are thus needed to properly respond to the emergent threat posed by *Vibrio* infections. The establishment and use of early warning systems for public health, such as the ECDC Vibrio Map Viewer platform developed by the European Centre for Disease Prevention and Control (ECDC) to monitor the environmental suitability of coastal waters for *Vibrio* spp. (Semenza et al., 2017) are also useful tools to face *Vibrio* infections increases in the 21st century due to climate change.

## CONFLICT OF INTEREST

The authors declare no conflict of interest.
